# Inflammatory mitochondrial nucleic acids as drivers of pathophysiology

**DOI:** 10.1002/ctm2.1403

**Published:** 2023-09-05

**Authors:** Christian G. Peace, Alexander Hooftman, Dylan G. Ryan

**Affiliations:** ^1^ School of Biochemistry and Immunology Trinity Biomedical Sciences Institute, Trinity College, Dublin Dublin Ireland; ^2^ Global Health Institute, Swiss Federal Institute of Technology Lausanne (EPFL) Lausanne Switzerland; ^3^ MRC Mitochondrial Biology Unit University of Cambridge Cambridge UK

1

Mitochondria are intracellular membrane‐bound organelles that harness the chemical energy in food to drive adenosine triphosphate (ATP) synthesis via oxidative phosphorylation. In the last several decades, mitochondria have emerged as central signalling hubs regulating cellular fate and function beyond their appreciated role in bioenergetics. This often requires the release of mitochondrial‐derived signals, such as cytochrome C, metabolites and reactive oxygen species, into the cytosol, all of which have been linked to regulating cell death, inflammation and cancer.^1^

**FIGURE 1:**
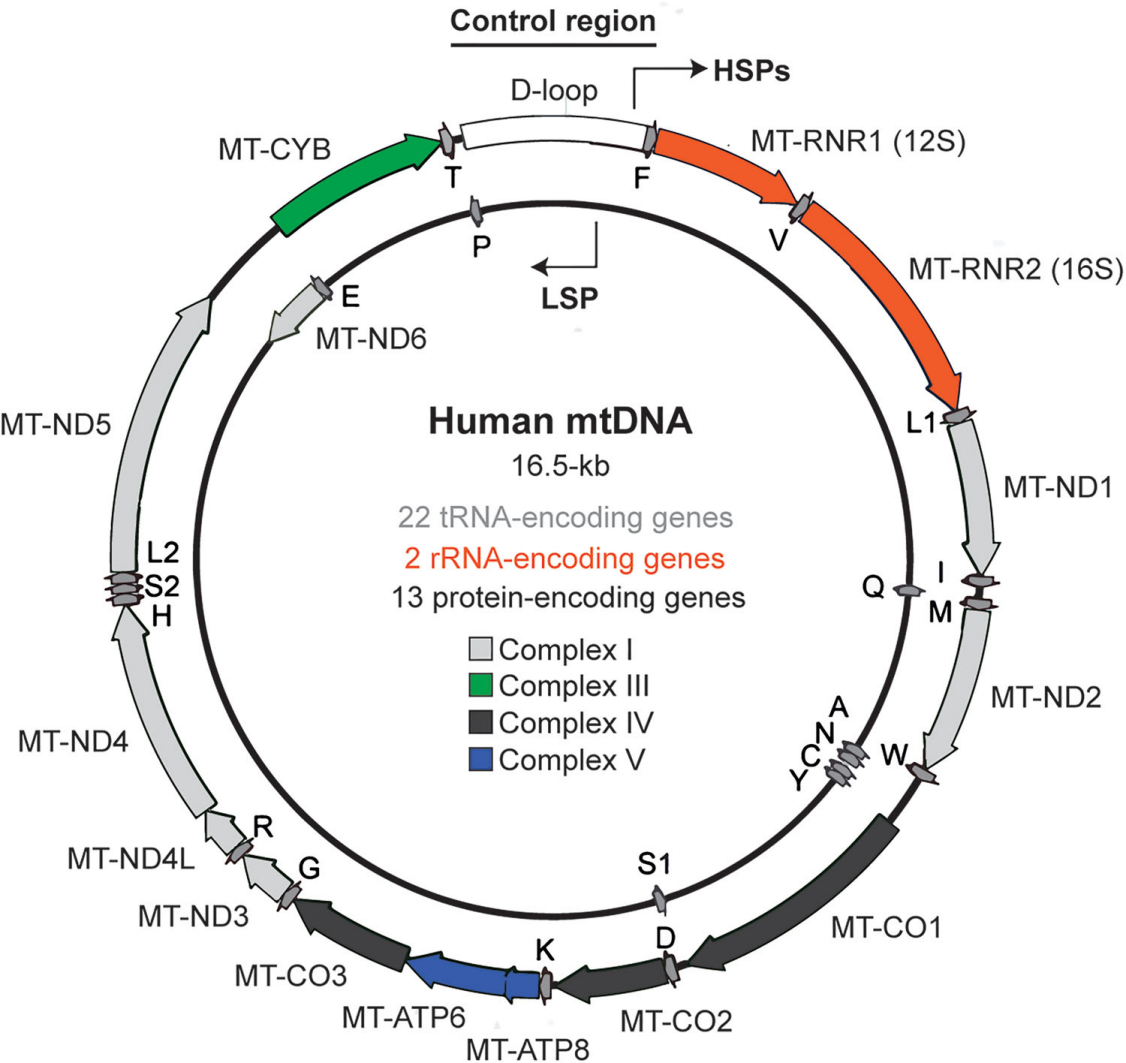
Schematic of human mitochondrial DNA.

While there are over 1000 genes encoding human mitochondrial proteins, mostly located in the nucleus, mitochondria have retained a circular 16569 base pair mitochondrial DNA (mtDNA) genome encoding 37 genes including 13 proteins that are critical components of the electron transport chain (ETC)^2^ (Figure 1). Within mitochondria, mtDNA is transcribed into mitochondrial RNA (mtRNA) which is translated into proteins by the mitochondrial ribosome. While the origins of mtDNA likely result from the endosymbiotic relationship between the alphaproteobacteria and archaeal host that gave rise to eukaryotic cells, it is still unclear why mitochondria retain a separate genome in most eukaryotic species given restricted mtDNA damage and replication quality control mechanisms. A potential explanation is that a reduced mitochondrial genome was retained in part to function as a means to communicate mitochondrial integrity to the rest of the cell.

As mitochondrial nucleic acids are highly immunostimulatory, these are strictly controlled and retained within the mitochondrial double endomembrane system. However, in numerous circumstances, mitochondria have been found to undergo programmed release of their nucleic acids to drive inflammatory signalling cascades through the activation of nucleic acid receptors such as cGAS, RIG‐I‐like receptors, and toll‐like receptors 3,7,8, which leads to interferon β release and antiviral signalling. Additionally, nucleic acid release also induces inflammasome activation triggering gasdermin D pore formation, pyroptosis, and interleukin‐1β release.

While early work on mitochondrial nucleic acids as drivers of inflammation focused predominantly on mtDNA, it has now become clear that mitochondria can release both single‐stranded (ss‐) and double‐stranded (ds‐) RNA under varying conditions. Programmed release of nucleic acids has been found to occur through Bak‐Bax‐mediated mitochondrial herniation, gasdermin pores localised to the mitochondrial outer membrane, the mitochondrial permeability transition pore, voltage‐dependent anion channel oligomerisation, and more recently, mitochondrial‐derived vesicles.^3^

**FIGURE 2:**
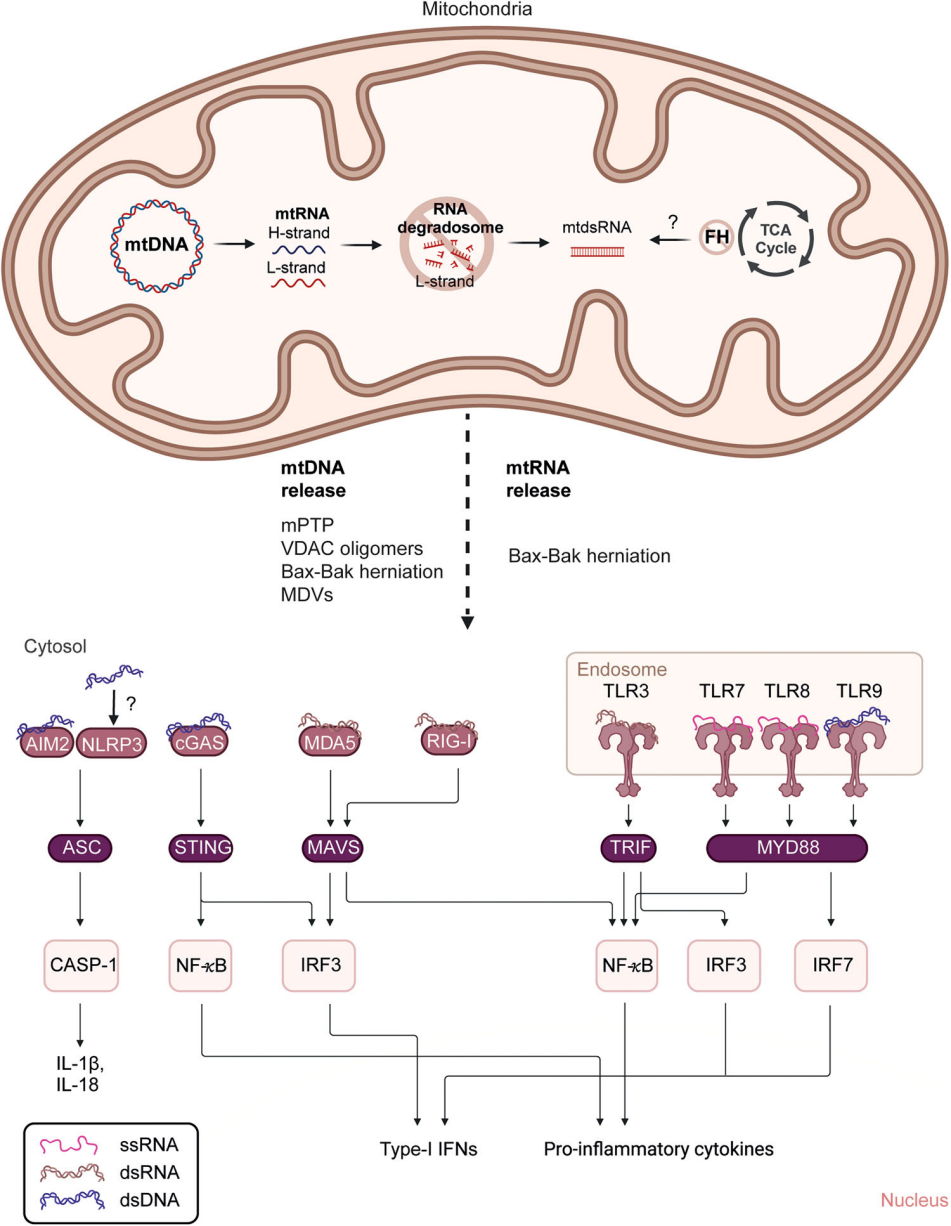
Mitochondrial nucleic acid sensing and release.

The role of dsRNA signaling in inflammatory diseases is beginning to emerge and may represent a novel mechanistic target for the treatment of these diseases. dsRNA is a particularly immunostimulatory nucleic acid. Host dsRNA can present an autoimmune threat as it is biochemically indistinguishable from foreign dsRNA derived from invading viruses and bacteria. Hence safeguarding mechanisms are required to prevent host dsRNA ligation of its cognate receptors, RIG‐I and MDA5 (Figure 2). Under homeostatic conditions, host dsRNA is contained within immune‐privileged organelles such as the mitochondrion and 99% of detectable cellular dsRNA is in fact of mitochondrial origin.^4^ Furthermore, the RNA degradosome functions in restricting the accumulation of dsRNA in the mitochondrion. Biallelic hypomorphic mutations in a member of the RNA degradosome, *PNPT1*, which encodes the exonuclease PNPase that dismantles dsRNA, lead to mitochondrial dsRNA (mtdsRNA) accumulation and a type I interferon (IFN) response in patients carrying these mutations,^4^ in addition to respiratory‐chain deficiency. While the observation that genetic inactivation of RNA‐degrading enzymes leads to an accumulation of mtdsRNA and subsequent immune response is not entirely surprising, can this process more broadly be governed by mitochondrial function and state? Furthermore, can a loss of mitochondrial integrity, and an associated release of mtdsRNA, play a role in the pathogenesis of more common inflammatory diseases?

We found that mtdsRNA does indeed accumulate following perturbation of mitochondrial metabolic enzymes, namely in the context of fumarate hydratase (FH) inactivation and inhibition of ATP synthase.^5^ Importantly from a clinical perspective, we found FH expression to be suppressed in whole blood samples of systemic lupus erythematosus (SLE) patients,^5^ where autoantibodies to dsRNA have previously been detected, indicating that FH suppression and associated impairment in mitochondrial bioenergetics may be drivers of disease pathogenesis in SLE. The importance of dsRNA sensing in the pathogenesis of inflammatory diseases is perhaps best illustrated by the fact that protective loss‐of‐function alleles in the dsRNA sensor MDA5 have been identified by genome‐wide association studies in a multitude of inflammatory diseases, including type I diabetes, psoriasis and inflammatory bowel disease.^6^ RNA editing is one mechanism by which autoimmunity can be avoided. dsRNAs can be rendered non‐immunogenic through the action of RNA‐editing enzymes such as ADAR1, and a reduced level of dsRNA editing is associated with an increased risk of multiple autoimmune diseases,^6^ indicating that the dsRNA‐RIG‐I/MDA5 axis contributes to the genetic risk of inflammatory disease. Finally, gain‐of‐function mutations in TLR7, which senses ssRNA and guanosine‐containing ligands, have been reported to cause SLE.^7^ TLR7 is also involved in driving the type I IFN response downstream of FH suppression.^5^

Given the growing body of evidence supporting a role for mtdsRNA in inflammatory disease pathogenesis, how and at what level may this pathway be targeted? Restoring mitochondrial bioenergetics, scavenging of cytosolic dsRNA and inhibition of dsRNA sensors may all be approaches that have therapeutic utility in this context. Activators of human FH have been described^8^ which could potentially improve mitochondrial bioenergetics in conditions where FH suppression has been reported, such as SLE. Similarly, high‐throughput screens have resulted in the development of potent small molecule antagonists of RIG‐I which are able to block type I IFN induction in vitro.^9^ Interestingly, mtRNA does not drive an IFN response in adipocytes isolated from young mice, in contrast to what occurs in adult adipocytes. Young adipocytes possess an endogenous safeguard mechanism due to low *Irf7* expression driven by vitamin‐D3 signalling. Vitamin‐D3 treatment of adult high‐fat diet‐fed mice induced similar repression of mtRNA‐driven inflammation,^10^ indicating that this could form the basis of dietary interventions to reduce dsRNA‐driven inflammation.

The clinical relevance of dsRNA‐driven inflammation has over time expanded from its implication in monogenic diseases to its role in more complex, common inflammatory diseases, and now seems ripe for exploration in our quest to develop novel anti‐inflammatory therapies. Therapeutics could target mtdsRNA accumulation, release, or downstream pathways. Future studies should work to delineate further molecular regulators of the pathway in order to identify potential clinical targets for intervention.
